# Neuropharmacology of human TERA2.cl.SP12 stem cell-derived neurons in ultra-long-term culture for antiseizure drug discovery

**DOI:** 10.3389/fnins.2023.1182720

**Published:** 2023-06-15

**Authors:** Hamed Salmanzadeh, Ankita Poojari, Atefeh Rabiee, Benjamin D. Zeitlin, Robert F. Halliwell

**Affiliations:** ^1^Thomas J. Long School of Pharmacy, University of the Pacific, Stockton, CA, United States; ^2^Arthur A. Dugoni School of Dentistry, University of the Pacific, San Francisco, CA, United States

**Keywords:** multi electrode array, anticonvulsants, neural networks, GABA_A_ receptors, ionotropic glutamate receptors, TERA2.cl.SP12 stem cells

## Abstract

Modeling the complex and prolonged development of the mammalian central nervous system *in vitro* remains a profound challenge. Most studies of human stem cell derived neurons are conducted over days to weeks and may or may not include glia. Here we have utilized a single human pluripotent stem cell line, TERA2.cl.SP12 to derive both neurons and glial cells and determined their differentiation and functional maturation over 1 year in culture together with their ability to display epileptiform activity in response to pro-convulsant agents and to detect antiseizure drug actions. Our experiments show that these human stem cells differentiate *in vitro* into mature neurons and glia cells and form inhibitory and excitatory synapses and integrated neural circuits over 6–8 months, paralleling early human neurogenesis *in vivo*; these neuroglia cultures display complex electrochemical signaling including high frequency trains of action potentials from single neurons, neural network bursts and highly synchronized, rhythmical firing patterns. Neural activity in our 2D neuron–glia circuits is modulated by a variety of voltage-gated and ligand-gated ion channel acting drugs and these actions were consistent in both young and highly mature neuron cultures. We also show for the first time that spontaneous and epileptiform activity is modulated by first, second and third generation antiseizure agents consistent with animal and human studies. Together, our observations strongly support the value of long-term human stem cell-derived neuroglial cultures in disease modeling and neuropsychiatric drug discovery.

## Introduction

Successful isolation and culture of human embryonic stem (ES) cells by Thomson et al. (1998) and later, induced pluripotent stem (iPS) cells by Yamanaka et al. (2007) gave rise to great excitement and optimism that they could be exploited in regenerative medicine, disease modeling and drug discovery (Gearhart, 1998; Gunaseeli et al., 2010; Hoang et al., 2022; Kim et al., 2022). Since these milestone discoveries, significant progress has been made delineating protocols and culture conditions to create specific tissues from ES and iPS cells, especially for different types of human neurons and glia, including hippocampal, hypothalamic, cortical, and spinal neurons, as well as for oligodendrocytes, astrocytes and microglia (Benchoua et al., 2021). Initial evaluation of neurons from human pluripotent stem cells were often limited to protein and gene expression data, along with morphological changes in the differentiated cells (Halliwell, 2017). More recent studies include electrophysiological data, which is essential in exploiting such human cells in drug discovery and safety evaluation for neuropsychiatric medicines (Halliwell, 2017; Sharlow et al., 2020; Jezierski et al., 2022).

Incorporation of pluripotent stem cell-derived neurons in drug screening has now been reported for a variety of genetic diseases such as Fragile-X Syndrome (Urbach et al., 2010; Sheridan et al., 2011), neurodegenerative diseases including Alzheimer’s disease (Arber et al., 2017) and psychiatric disorders such as depression and schizophrenia (reviewed by Benchoua et al., 2021). Stem cell derived neurons utilized in such studies, however, are relatively immature and maintained in culture for only short periods of time (often days to a few weeks) and may not always represent mature neurons which can take many months to achieve (Halliwell, 2017; Sharlow et al., 2020). For example AMPA receptor subunit composition and single channel conductance change from an immature to a mature phenotype over 5 weeks in cultured excitatory cortical neurons derived from human ES cells (Livesey et al., 2014). Moreover, changes in the relative expression of the NKCC1 and KCC2 transporters take over 7 weeks *in vitro*, leading to a reduction in the intracellular chloride ion concentration and is the reason that GABA acts as an excitatory transmitter in *immature* neurons and an inhibitory transmitter in *mature* neurons (Livesey et al., 2014). Johnson et al. (2007) also elegantly demonstrated that human ES (H9) cells were only sufficiently mature to be able to fire sustained (TTX-sensitive) trains of action potentials by 10 weeks *in vitro* and this correlated with increased expression of the transient I_A_ current (which facilitates faster repolarization) and with the development of glial cells in the cultures (Johnson et al., 2007). The optimal time window to utilize stem cell derived neurons *in vitro* for studies of the site and mechanism(s) of drug action, and for drug screening and safety evaluation is therefore unclear (Sharlow et al., 2020; Tukker and Westerink, 2021). Moreover, the activity and neuropharmacological properties of human neurons in ultra-long-term culture, as they differentiate from stem cell to immature and subsequently mature neurons and later synaptically linked circuits and neural networks has not been extensively addressed.

Human germ cell tumors (GCTs) contain within their mass pluripotent stem cells (PSCs) which have given rise to several valuable clonal stem cell lines, including NTERA2 and GCT27 (Andrews, 2021). These embryonal carcinoma (EC)-derived stem cells are considered the malignant counterpart of human ES cells and share similar characteristics to ES cells and iPS cells (Andrews et al., 2005; Andrews, 2021). In the present study therefore, we have used TERA2.cl.SP12 cells, a pluripotent human EC stem cell line that undergoes neural differentiation when exposed to the morphogen, retinoic acid and forms both functional neurons and glial cells *in vitro* (Przyborski et al., 2000, 2004; Andrews, 2002; Stewart et al., 2004; Coyne et al., 2011). Specifically, we have utilized microscopy, immunocytochemistry and immunoblotting to identify neurons and glia together with multi-electrode arrays (MEAs) to record spike and burst firing from individual cells, as well as synchronized firing across synaptically connected networks of human stem cell derived neurons in ultra-long-term (≥1 year) cell culture. Moreover, we have addressed the development and progression of endogenous electrochemical activity and the neuropharmacological properties of neurons from the start of differentiation over the course of 1 year *in vitro*. Additionally, we have investigated a chemically and pharmacologically diverse selection of antiepileptic agents that act via distinct receptors and ion channels on spontaneous and induced epileptiform-like activity to determine the sensitivity, reliability and validity of such human stem cell-derived neuronal models for drug evaluation and discovery. To our knowledge, this is also the first study to address the impact of representatives of first, second and third generation antiseizure agents (Löscher and Klein, 2021) on both *young* and *mature* stem cell derived human neurons maintained *in vitro* over the course of a year.

## Materials and methods

### Cell culture

Human TERA2.cl.SP12 stem cells (kindly provided by Professor SA Przyborski, University of Durham; Przyborski, 2001) were maintained in a media consisting of Dulbecco’s Modified Eagles Medium (DMEM, Sigma–Aldrich), 10% Fetal Bovine Serum (FBS, Gibco), 2 mM L-glutamine (Gibco) and penicillin–streptomycin (100 U/ml, 100 μg/ml; Invitrogen) at 37°C, 5% CO_2_, 100% relative humidity in T-75 mL tissue culture flasks until they were 80% confluent. Cells were then removed by rolling micro-glass beads (Fisher Scientific) across the surface of the flask to detach them and re-seeded on pre-coated glass coverslips or MEA plates. Retinoic acid (10 μM; Sigma-Aldrich) was added to the culture media to differentiate stem cells toward neuronal phenotypes. The culture media was then refreshed every 2–3 days.

### Immunocytochemistry

Stem cells and neural derivatives were cultured at a density of 50,000 cells/cm^2^ on 18 mm diameter sterile glass coverslips pre-coated with poly-d-lysine (50 μg/ml; Sigma-Aldrich). Cells were rinsed in Phosphate Buffered Saline (PBS), fixed with 4% formaldehyde in PBS, washed again with PBS and permeabilized in 0.3% Triton X-100 in PBS at room temperature for 10 min. Cells were then washed and placed in blocking solution containing 5% normal donkey serum in PBS at room temperature for 1 h. Thereafter, cells were placed in dilutions of the primary conjugated antibodies (Santa Cruz Biotechnology) for Oct3/4 (1:100), βIII tubulin (1:100) or glial fibrillary acidic protein (GFAP, 1:100) in blocking solution, and incubated at 4°C overnight. Cells were then washed with PBS and counter-stained with DAPI (100 ng/ml; Sigma-Aldrich) before mounting over chamber slides using anti-fade mounting solution (Prolong1 Gold Antifade Reagent; Invitrogen). Immunolabeled cells were visualized through an inverted EVOS FL auto microscope (Thermo Fisher Scientific). Fluorescence was observed using filter sets appropriate for each label. The percentage of immunofluorescent cells was determined by counting DAPI^+^ cells and immuno-positive for βIII Tubulin and MAP2 in 5 fields (0.23 mm^2^ per field) across the slide.

### Immunoblotting

Cells were washed with PBS and lysed in RIPA lysis buffer (Sigma-Aldrich), followed by 2 min low intensity sonication and centrifugation for 15 min at 13,000 rpm. Protein concentrations were determined using Qubit protein assay kit (Thermo Fisher Scientific). Lysate was denatured by adding 25% 4x Laemmli Sample Buffer (Alfa Aesar) and 50 mM dithiothreitol (DTT), followed by heating at 95°C for 5 min. Equal amounts of cleared lysates were separated using SDS-PAGE and transferred to polyvinylidene difluoride (PVDF) membranes (Millipore). Non-specific binding sites were blocked by incubating the membranes in premade blocking buffer (Thermo Fisher Scientific). Afterwards, the membranes were incubated with primary antibodies of βIII-Tubulin (Santa Cruz Biotechnology) and GFAP (Santa Cruz Biotechnology) at 1:1000 concentration overnight at 4°C. After washing, HRP-conjugated secondary antibody of IgG (Santa Cruz Biotechnology) was applied at 1:1500 ratio for 1 h at room temperature. Blots were washed and incubated in enhanced chemiluminescence reagents (Millipore) and analyzed with Odyssey FC imaging system (Li-Cor Bioscience).

### Multi-electrode array recording

Recording of spontaneous activity was conducted once a week using the Maestro system and AxIS software (Axion Biosystems) over 1 year and replicated over a second year. A total of six, 6-well MEA plates contributed to the MEA data set. Each well contained an array of 64 PEDOT electrodes arranged in an 8 × 8 grid covering a recording area of 2.1 × 2.1 mm; each 6-well plate therefore has a total of 384 electrodes. Stem cells were plated at 200,000 cells on each well of the 6-well MEA plates (Cytoview MEA, Axion Biosystems) pre-coated with polyethyleneimine (Sigma-Aldrich) and maintained at 37°C, 5% CO2, 100% relative humidity. To sustain cultures, 50% of the media was replaced with fresh pre-warmed media every 3 days. All recordings were performed at a constant temperature of 37°C and 5% CO_2_. The MEA plates were placed into the Maestro Edge platform and equilibrated for 10 min prior to a baseline recording period. Electrical signals were sampled at 12.5 kHz, with bandwidth filters set between 200 Hz and 3 KHz; the spike threshold was computed with an adaptive threshold of 6 times the estimated rms noise on each channel. The RAW data files were re-recorded with AXIS software to convert the electrophysiological data into Microsoft Excel files including spike timing and profile information. Only electrodes with activity of at least 5 spikes per minute over the recording time were included for data analysis. The following parameters were calculated using AxIS Navigator software from the MEA data:

1. *Number of Active Electrode:* is the number of electrodes with activity of ≥5 spikes/min.
2. *Weighted Mean Firing Rate*: is the mean firing rate from only ‘active’ electrodes.
3. *Single Electrode Bursts*: are clusters of at least 5 spikes, with a maximum inter-spike interval (ISI) of 100 ms.
4. *Network Bursts*: are a minimum of 50 spikes, with an ISI of ≤100 ms and at least one third of the electrode array participating in a 20 ms time window.
5. *Synchrony Index*: is derived from the cross-correlogram of spikes occurring at times relative to each other across all unique pairs of electrodes. Values are between 0 and 1, with those closer to 1 indicating higher synchrony and coordinated neural activity (Halliday et al., 2006).

### Drug solutions

Stock solutions of drugs were freshly prepared and filtered using 0.22 μM solvent resistant filters (Millipore) as follows: topiramate, picrotoxin and tiagabine (Sigma-Aldrich), retigabine, XE991 (both from Alamone) and kynurenic acid (Tocris) were dissolved in DMSO to obtain stock solutions of 10 mM. Bicuculline, d-2-Amino-5-phosphonovaleric acid (D-AP5), 4-Aminopyridine (4-AP), phenobarbital, valproic acid (all from Sigma-Aldrich), 6,7-dinitroquinoxaline-2,3-dione (DNQX), tetrodotoxin (TTX) (both from Tocris) were dissolved in water to obtain stock solutions of 10 mM. Carbamazepine, ethosuximide and diazepam (all from Sigma-Aldrich) were dissolved in ethanol to obtain stock solutions of 10 mM. All drugs were then serially diluted to their final concentrations in cell culture/recording media.

### Drug testing procedures

Recordings of spontaneous activity were obtained for at least 30 min before any drug treatment and again for a minimum of 30 min in the presence of one of the following control ion channel or receptor agonists or antagonists added to the cell media: the sodium channel blocker, tetrodotoxin (10 nM), the potassium channel activator, retigabine (3 μM), the potassium channel inhibitor, XE991 (3 μM), the GABA_A_ receptor antagonist bicuculline (10 μM), the GABA_A_ receptor potentiator, phenobarbital (100 μM), the AMPA/kainate receptor antagonist, DNQX (10 μM), the NMDA receptor antagonist D-AP5 (30 μM) or the broad spectrum glutamate receptor antagonist, kynurenic acid (100 μM). At the end of each drug experiment, the culture media was removed and replenished three times. Two or three days later, 90% of the culture media in each well was again replenished with fresh media. To avoid possible residual effects of drug exposure, pharmacological testing was carried out at intervals of at least 2 days and frequently at intervals of weeks.

To evaluate the reliability and validity of human stem cell-derived neurons for drug exploration, we conducted pilot experiments and determined that 4-AP (100 μM) reliably increased spike activity and triggered burst firing from individual neurons as well as network burst and synchronized neural network activity, typical of *epileptiform* activity *in vitro* (Tukker and Westerink, 2021). Control spontaneous firing was therefore recorded for a minimum of 10 min, 4-AP was then added, and activity recorded for a further 60 min. This was followed by addition of one of the following antiseizure drugs: carbamazepine (10 μM), topiramate (100 μM), diazepam (10 μM), ethosuximide (100 μM) or tiagabine (30 μM), and the MEA recording was continued for a further 40–50 min. All drugs were washed out once an asymptotic change had been established and the cells replenished with fresh culture media. They were then placed back into the CO_2_ incubator. Our pharmacological experiments were conducted on cells differentiated between 8 and 52 weeks *in vitro*.

### Statistical analysis

All statistical tests were conducted using GraphPad Prism software (Version 9). Data was analyzed with Paired or Unpaired, two-tailed Student’s *t-*tests, One-Way Analysis of Variance (ANOVA) with Tukey post-hoc tests, or Kruskal-Wallis with Dunn post-test corrections. Statistical significance was set at *p* ≤ 0.05, and individual *p-*values are indicated in the legend of each graph.

## Results

### Microscopy and immunocytochemistry analysis

**Figure 1:**
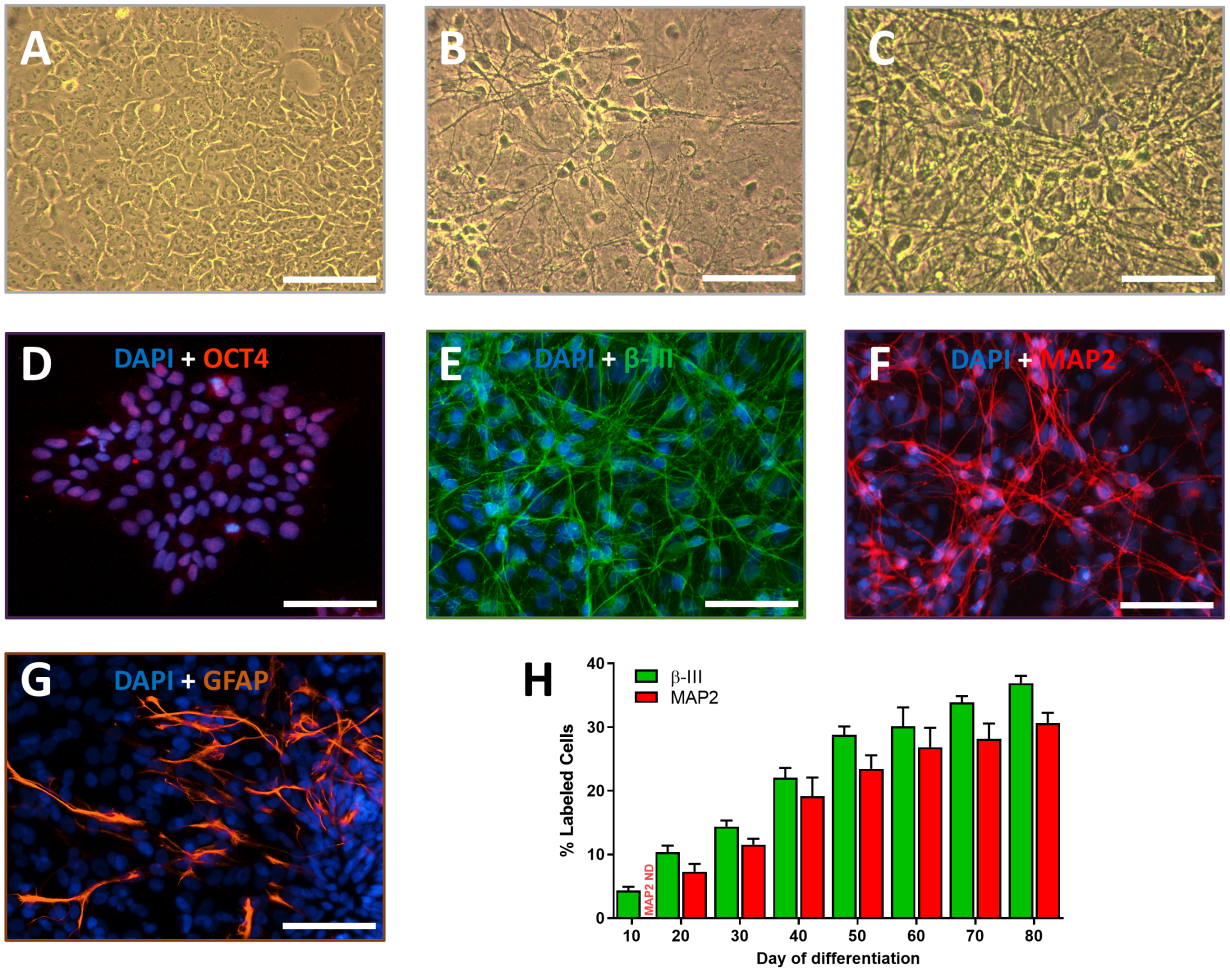
Neural differentiation of human TERA2.cl.SP12 stem cells: Phase contrast images show **(A)** undifferentiated stem cells in culture, **(B)** neurons derived from them at 30 days, and **(C)** 80 days differentiation with retinoic acid. Immunofluorescence images show cells fixed and stained with the nuclear dye DAPI (blue) plus **(D)** Oct-4 (red) prior to differentiation; **(E)** β-III tubulin (green); **(F)** MAP2 (red), or **(G)** glial-fibrillary acidic protein (GFAP, orange) at 80 days neural differentiation. All scale bars = 100 μm. **(H)** The histogram shows the percentage of DAPI stained cells that were also β-III tubulin^+^ or MAP2^+^ cells (on the *y*-axis) over 80 days differentiation (*x*-axis). The bars and lines represent the mean ± SEM (*n* = 3–5).

Neurons and glial cells were successfully derived from TERA2.cl.SP12 by exposure to retinoic acid (10 μM). Differentiated cells were well maintained as confluent monolayers, adherent to MEA plates or glass coverslips coated with PEI or poly-d-lysine, respectively for up to 12 months *in vitro*. The characteristics of individual neurons in the cultures, such as polarized cell bodies with neurite network formation and phase-bright perikaryon became identifiable among populations of non-neuronal cells around 20 days differentiation. The neurons were phase-bright with spherical or pyramidal cell bodies and multiple neurite processes that became more elaborate and denser over time *in vitro* (Figures 1A–C). Immuno-staining showed non-differentiated cells expressed the pluripotent stem cell markers Oct-4 (Figure 1D) and SOX2 (not shown) and labeled with the DNA dye, DAPI. Oct-4 labeling of stem cells was no longer detected 5 days after the start of neural differentiation with retinoic acid. Immunolabeling with the neuronal markers, β-III tubulin and MAP2 (two microtubule proteins found in neurons) was determined over the first 3 months of neural differentiation and increased from approximately 5% at day 10 to almost 40% (for β-III) at day 80 *in vitro* (Figures 1E,H), consistent with previous studies (Cao et al., 2015). MAP2^+^ cells (Figures 1F,H)were not detected until day 20 of neural differentiation *in vitro,* consistent with reports that MAP2 is a marker of intermediate neuronal maturation (Soltani et al., 2005). Glial cells, indicated by the expression of the astrocyte marker GFAP (Figure 1G), were not detected before day 50 day of neural differentiation. However 9 ± 1% (*n* = 5) of DAPI^+^ cells labeled for GFAP at day 80 and this increased to 15 ± 3% (*n* = 3) by day 120 differentiation, indicating that gliogenesis follows neurogenesis in these human stem cell cultures, paralleling neural development *in vivo* and following an intrinsic developmental clock (Kriegstein and Alvarez-Buylla, 2009) even *in vitro*.

### Immunoblotting

**Figure 2:**
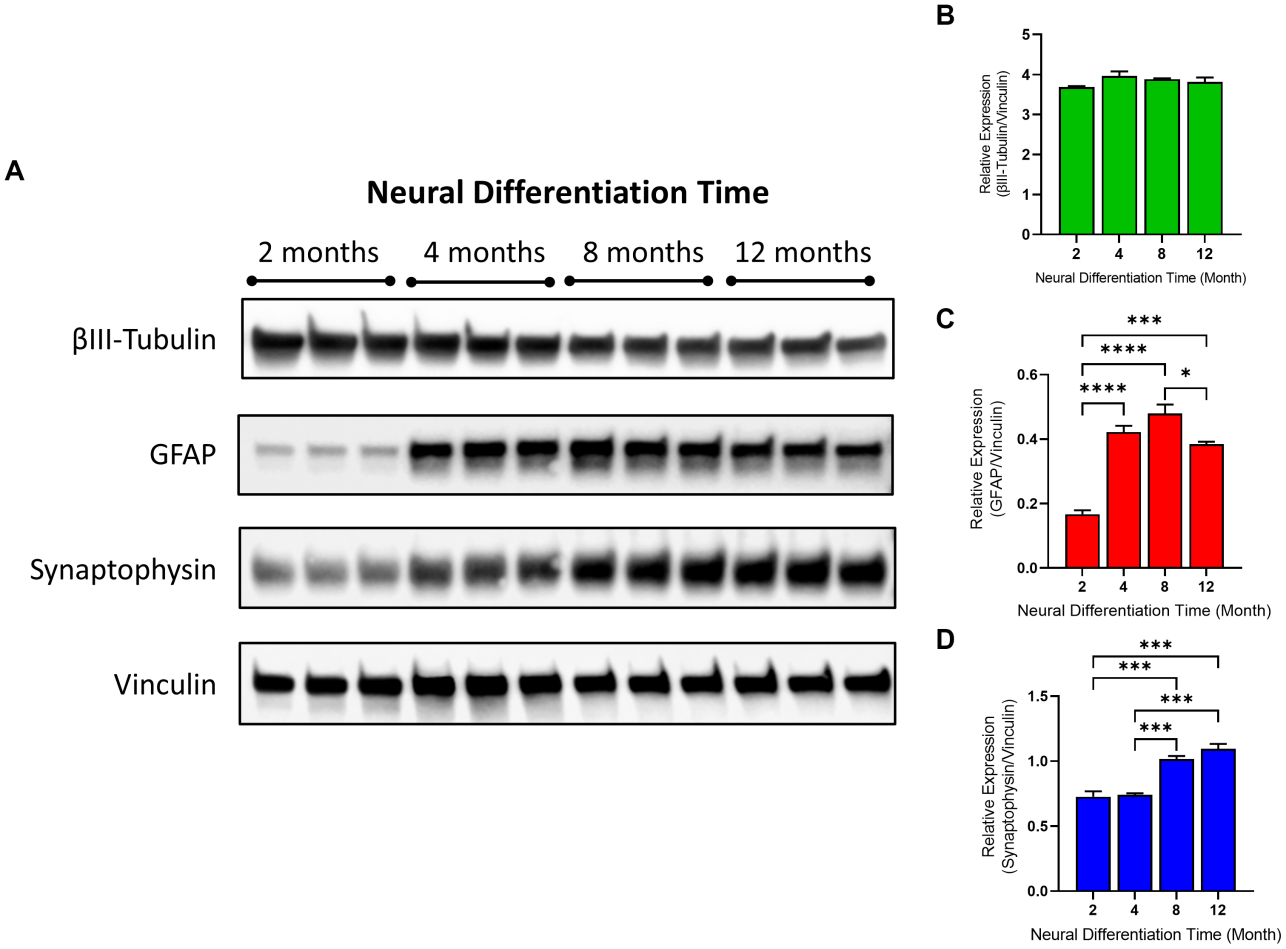
Protein expression and analysis of neuroglial markers in human TERA2.cl.SP12 stem cell derived neurons and glia over 1 year *in vitro*. **(A)** Shows immunoblots of the expression of β-III Tubulin, GFAP, synaptophysin and vinculin proteins at 2, 4, 8, and 12 months, in triplicate, at each time point. The histograms show the relative protein expression of **(B)** β-III tubulin to vinculin **(C)** GFAP to vinculin and **(D)** synaptophysin relative to vinculin. Statistical analysis of the experimental groups was determined using one-way ANOVA followed by Tukey’s post-test. Values are the mean ± SEM (*n* = 3); ∗*p* ≤ 0.05, ***p ≤ 0.001, ****p ≤ 0.0001.

The expression of the neural protein, β-III tubulin, the glial cell protein, GFAP and the synaptic protein, synaptophysin, relative to the loading control protein, vinculin were determined at 2, 4, 8, and 12 months from the start of neural differentiation. β-III tubulin was well expressed in the cultures and remained consistent from 2 to 12 months. In contrast, GFAP expression was low at 2 months (consistent with our immunolabeling data) but increased significantly by 2.5-fold at 4 months and by 2.8-fold at 8 months and remained over 2.2 times higher at 12 months. Notably, synaptophysin expression was low at 2 and 4 months but significantly increased at 8 and 12 months of neural differentiation (see Figure 2).

### Multi electrode array recorded activity

**Figure 3:**
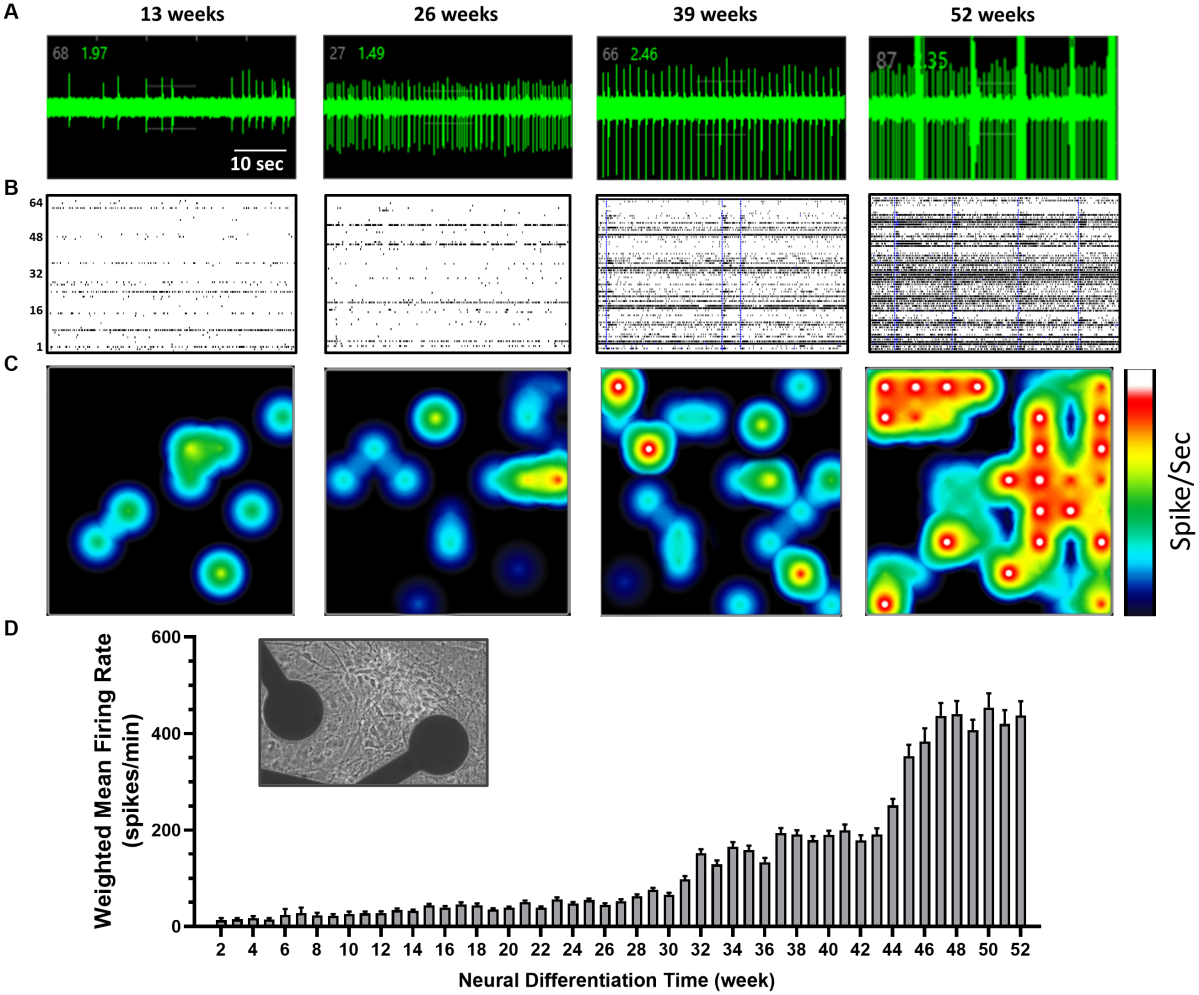
Multi-electrode array (MEA) recordings from human TERA2.cl.SP12 stem cell-derived neurons over 1 year in culture: **(A)** shows action potentials as individual spikes over baseline noise at 13, 26, 39, and 52 weeks of differentiation. Note in this cell the increasing frequency and amplitude of spikes and rhythmical bursting seen at 52 weeks **(B)** shows a Raster plot of spike frequency, represented by the lines and detected by the electrode array (numbered 1–64 on the left). Clusters of lines indicate cell bursts, and the blue vertical lines indicate network bursting. **(C)** Shows a color-coded heatmap with a snapshot from a 6 well plate with blue indicating lower (*circa* 1 Hz) spike frequency and red/white higher (10 Hz) individual cell firing rates. Note that over time, increased connectivity of cell activity across this single well. **(D)** A histogram summary of the time course of mean ± SEM (*n* = 23) firing rates per active electrode from week 2 to 52. The inset shows a phase-contrast image of cells on an MEA plate (with 2 of the 64 electrodes per well seen in this image) taken at 1 year *in vitro*.

*S*pontaneous electrical activity was successfully recorded from stem cell-derived neurons cultured on MEA plates over 52 weeks of differentiation. For our initial characterization we limited our analysis to the weighted mean firing rates. Sparse and occasional spikes were observed approximately 2–3 weeks after the start of differentiation and increased significantly over 1 year *in vitro* from 28 ± 3 spikes/min (≤0.5 Hz) at 12 weeks to reach a plateau around week 48 with a mean firing rate of 439 ± 27 spikes/min (around 7 Hz). Figure 3 shows an extracellular spike recording from an individual unit at 13, 26, 39 and 52 weeks, a Raster plot of spike activity from all 64 electrodes in one of the 6 wells in an MEA plate and a heat map from a single well over the same time points, all illustrating the enormous development of neural activity in these cultures over 1 year.

**Figure 4:**
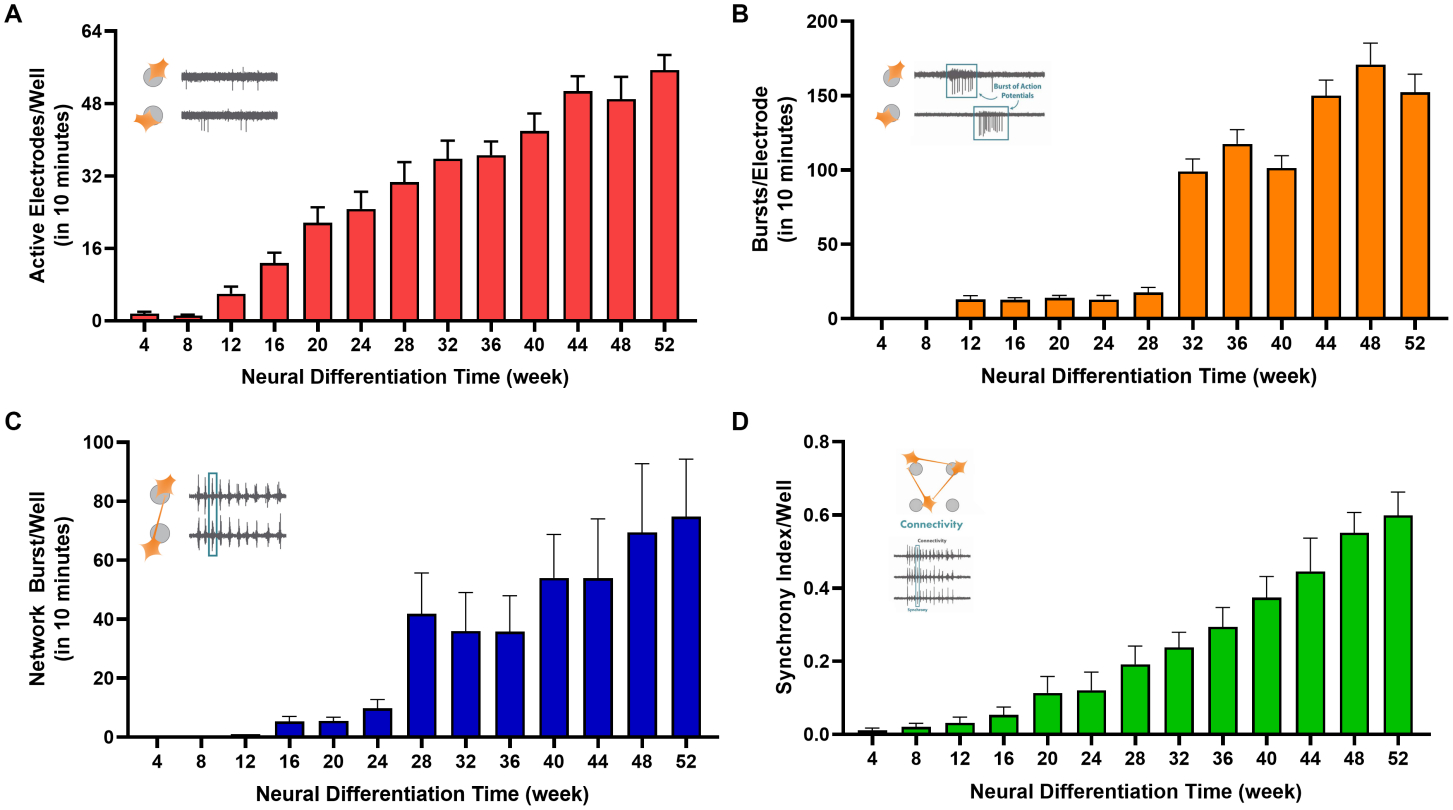
Electrophysiological properties of human TERA2.cl.SP12 stem cell-derived neurons differentiated over 1 year *in vitro*: the histograms show **(A)** the average number of active electrodes per well; **(B)** the average number of bursts recorded per electrode; **(C)** the average number of network bursts per well; and **(D)** the mean synchrony index per well. The increase in all measured parameters over the year in cell culture. The bars and lines represent the mean ± SEM from 6 MEA plates. The inset in each graph represents the parameter measured.

In addition to the significant increase in spike activity over the course of 1 year, the number of *active* electrodes in each well increased from an average of 1.6 ± 0.4 electrodes at week 4, to 55.4 ± 3.3 of the 64 recording electrodes per well at week 52 (Figure 4A). This observation is consistent with an increasing number of differentiated and functionally mature neurons in the cell cultures.

Single cell burst firing began at week 12 with 12 ± 2 bursts/10 min (n = 24) recorded and increased significantly to 152 ± 12 (n = 24) by week 52 of differentiation with a 5-fold increase between 28 and 32 weeks (see Figure 4B). Network busting also greatly increased at week 28 of neural differentiation (Figure 4C). Action potentials within bursts were generated at frequencies up to 80 Hz by week 20. Coordinated neural firing, indicated by the synchrony index, was essentially absent up to week 8 but increased markedly thereafter, coinciding with the emergence of glial cells in the cultures (see Figure 4D). These data show the development and physiological maturation of functional human neurons and coordinated firing through connected neural circuits. Synchronized neural network activity is critical in the development of the nervous system and is observed in the cerebral cortex of premature human fetuses around 20 weeks of gestation (Khazipov and Luhmann, 2006).

### Neuropharmacology of stem cell derived neuron activity

**Figure 5:**
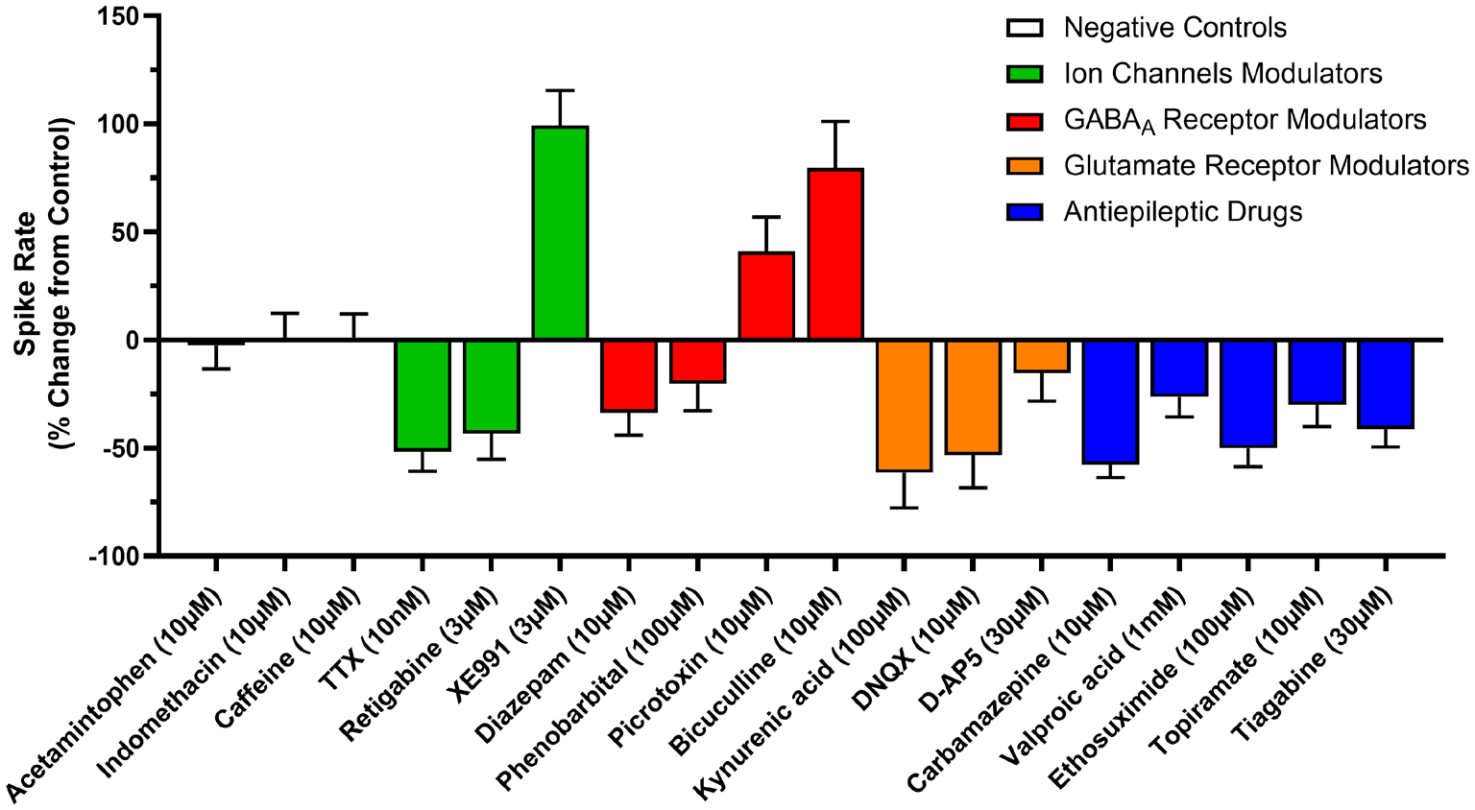
Modulation of cell and circuit activity recorded from human TERA2.cl.SP12 stem-cell derived neurons: the graph shows changes in spike rate (*y*-axis) from control induced by a variety of drugs (*x*-axis) acting at voltage or ligand-gated channels, or via actions on synaptic neurotransmission, determined between 16- and 20-weeks neural differentiation. TTX and retigabine reduced activity, as did the GABA_A_ potentiators, diazepam and phenobarbital, the ionotropic antagonists (DNQX, D-AP5 and kynurenic acid) and the antiseizure agents (valproic acid, carbamazepine, ethosuximide, topiramate, and tiagabine). In contrast, the two GABA_A_ antagonists, picrotoxin and bicuculline, and the potassium channel blocker, XE991 increased spike rates. Acetaminophen, indomethacin and caffeine had no effect. (0 = no change; +% = increased spike rate; −% = reduced spike rate). The bars and lines represent the mean ± SEM from *n* = 3–4 experiments.

Neuronal spiking, burst firing and coordinated network activity involves a complex array of ion channels and synaptic receptors in the brain. We therefore determined the impact of a diverse group of voltage and ligand-gated ion channel modulators on the human stem-cell-derived neural activity. Spontaneous spike activity was reduced by 51 ± 9% (*n* = 6) in the presence of the sodium channel blocker tetrodotoxin (TTX, 10 nM), and by 43 ± 12% (*n* = 6) by the potassium (Kv7) channel modulator, retigabine (3 μM). In contrast, the Kv7 channel blocker, XE991 (3 μM) increased firing by 99 ± 16% (*n* = 6) (see Figure 5). Together these data confirm that the MEA recorded activity was the result of sodium and potassium-dependent action potentials in our neuroglia cultures and is consistent with patch-clamp recordings from these cells (Coyne et al., 2011).

In the mammalian brain, GABA_A_ and ionotropic glutamate receptors mediate fast inhibitory and excitatory synaptic transmission, respectively (Samardzic, 2018). The GABA_A_ receptor antagonist, bicuculline (10 μM) and the GABA_A_ channel inhibitor picrotoxin (10 μM) increased the spontaneous spike rate by 79 ± 21% (*n* = 6–9) and 41 ± 16%, (*n* = 6–9) respectively. In contrast, the positive allosteric modulators of the GABA_A_ receptor, diazepam (10 μM) and phenobarbital (100 μM) reduced the spike rate by 34 ± 10% (*n* = 6–9) and 20 ± 13% (*n* = 6–9), respectively. Addition of the broad-spectrum glutamate receptor antagonist, kynurenic acid (100 μM) reduced the spike rate by 61 ± 16% (*n* = 6–9); similarly, the AMPA receptor selective antagonist DNQX (10 μM) and the NMDA receptor selective antagonist D-AP5 (30 μM) reduced the spontaneous spike rate by 80 ± 11% and 53 ± 15%, (*n* = 6) respectively (see Figure 5). Together, these data indicate that our cultures are composed of a heterogeneous population of both excitatory and inhibitory neurons, with synaptic transmission largely mediated by GABA_A_ and ionotropic glutamate receptors. These long-term neuroglial cultures therefore display several major hallmarks of complex neural networks observed *in vivo*, including neurons capable of generating sustained high frequency action potential discharge, network bursting, synchronized firing, and both inhibitory and excitatory neurotransmission that is sustained for at least 1 year *in vitro*.

### Impact of neuroglia differentiation age *in vitro* on drug sensitivity

The optimal age (or maturity) of human stem cell derived neurons in culture for drug studies is unclear (Sharlow et al., 2020; Tukker and Westerink, 2021). We therefore addressed the impact of the GABA_A_ antagonist, bicuculline and the potentiator (and anticonvulsant), phenobarbital, the AMPA antagonist, DNQX, the NMDA antagonist D-AP5, and the antiseizure agent, valproic acid on spontaneous neuronal spike rates at 8–10 weeks, 16–20 weeks and 40–45 weeks neural differentiation. Bicuculline (10 μM) increased spontaneous activity by approximately 40% at 8–10 weeks and by around 70% at the two later time points. Conversely, phenobarbital (100 μM) reduced spiking by 22% at 8–10 weeks, 30% by weeks 16–20, and approximately 40% at weeks 40–45 neural differentiation. Similarly, DNQX (10 μM) and D-AP5 (30-100 μM) each reduced spontaneous activity by *circa* 20% at 8–10 weeks but by 40–50% at the two later time points. Together, these data indicate an increased number of active GABA_A_ and ionotropic glutamate receptor mediated events in older and neurophysiologically mature cultures (Table 1). In contrast, valproic acid (1 mM) reduced spike activity by approximately 20% at all 3 time points (Table 1). These data show that drug actions can be detected reliably in maturing and highly established human stem cell deri*ved neuroglial* cultures. We next addressed their sensitivity to a pharmacologically diverse array of five antiepileptics to address the value of our neuroglial cultures in neurological drug discovery.

**Table 1:** Summarizes the effects of two GABA_A_ and two ionotropic glutamate acting drugs, and the second-generation anticonvulsant, valproic acid on spike rate recorded from TERA2.cl.SP12 stem cell-derived neurons at three time periods of neural differentiation.

Drug	Week 8–10	Week 16–20	Week 40–45
Bicuculline (10 μM) *N* = 3–6	39%↑ (±5)	78%↑ (±10)	67%↑ (±12)
Phenobarbital (100 μM) *N* = 3–6	22%↓ (±8)	31%↓ (±12)	39%↓ (±11)
DNQX (10 μM) *N* = 3–6	19%↓ (±9)	40%↓ (±10)	44%↓ (±12)
D-AP5 (100 μM) *N* = 3–6	21%↓ (±6)	51%↓ (±13)	49%↓ (±9)
Valproic acid (1 mM) *N* = 3–6	23%↓ (±8)	26%↓ (±9)	18%↓ (±11)

Note the impact of the GABA_A_ antagonist, bicuculline and the GABA_A_ potentiator, phenobarbital increases over time in culture. Similarly, the effects of the two glutamate receptor antagonists also increased as the cultures mature, whereas the impact of valproic acid remained consistent. These data suggest an increased number of active inhibitory and excitatory synapses mediating neural activity. The spike rate values are expressed as a mean % of control (±SEM, *n* = 3–6) with ↑ indicating an increase and ↓ a decrease in spike rate.

### Neural activity and antiseizure drug actions

Carbamazepine, valproate, and topiramate are widely used in seizure control, mood stabilization and migraine prophylaxis; ethosuximide is a first line agent for *absence* epilepsy and tiagabine is an add-on agent for refractory seizure control (Hakami, 2021). These agents have distinct mechanisms of action which includes inhibition of voltage-gated Na channels (carbamazepine), inhibition of T-type calcium channels (ethosuximide), GABA reuptake block (tiagabine) or more complex sites of action (topiramate and valproate, Sills and Rogawski, 2020; Hakami, 2021). All these antiseizure agents reduced spontaneous firing as follows: carbamazepine (30 μM) by 57 ± 6% (*n* = 3–6); valproic acid (1 mM) by 26 ± 9% (*n* = 3–6); ethosuximide (100 μM) by 50 ± 8% (*n* = 3–6); topiramate (10 μM) by 30 ± 10% (*n* = 3–6); and tiagabine by 41 ± 8% (*n* = 3–6) (see Figure 5). In contrast, no change in spontaneous activity was found when cells were exposure to the control drugs, caffeine (10 μM), acetaminophen (10 μM) or indomethacin (10 μM).

### Epileptiform activity and antiseizure drug efficacy

**Figure 6:**
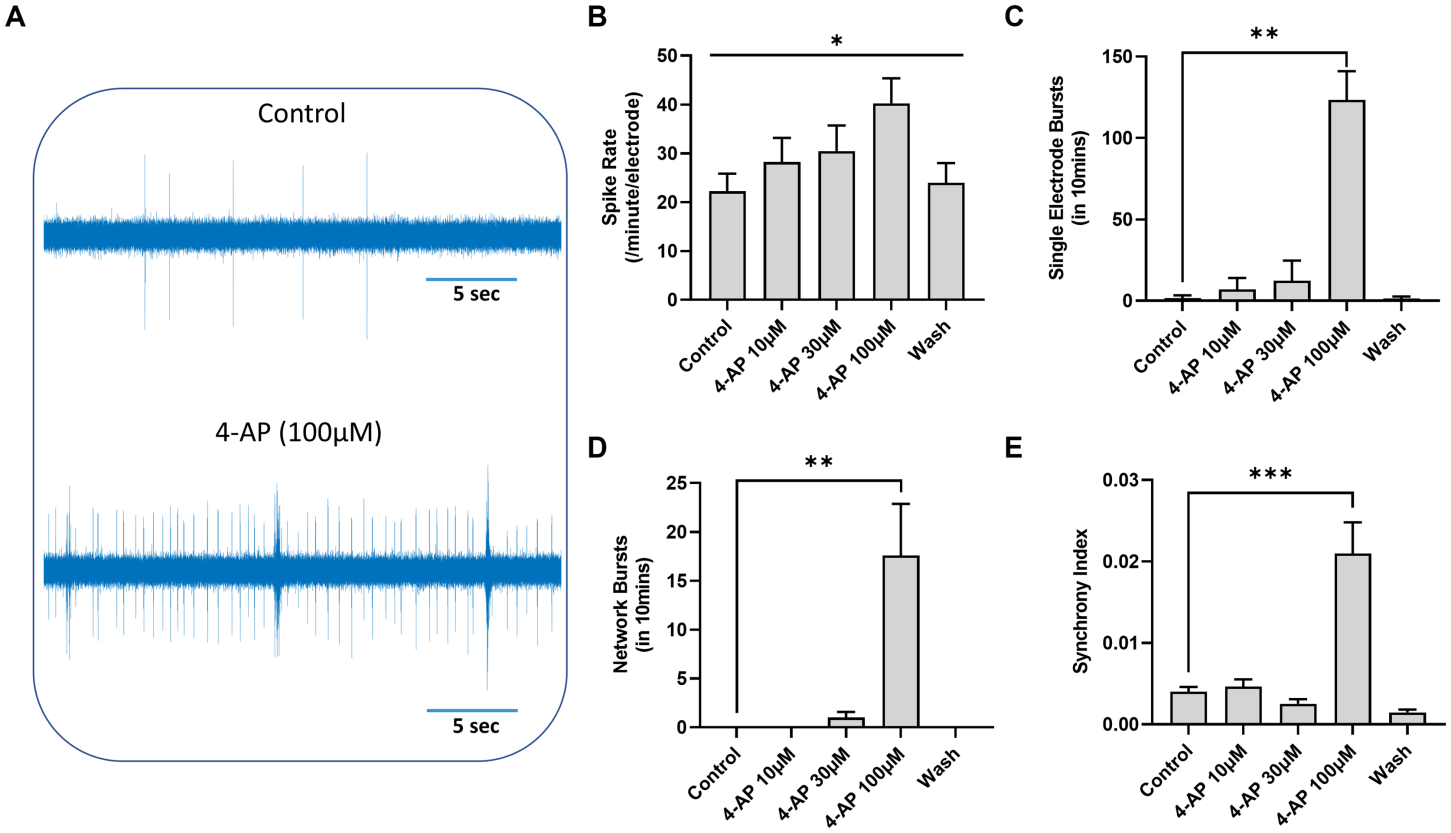
4-Aminopyridine (4-AP) induced epileptiform-like activity in human TERA2.cl.SP12 stem cell derived neurons: **(A)** shows an extracellular recording from one MEA channel of spontaneous spike activity before (top) and after (bottom) addition of 4-AP (100 μM) to the well. **(B)** A histogram showing that 4-AP (10–100 μM) dose-dependently increased spontaneous firing rates and **(C)** at 4-AP (100 μM) significantly increased the number of individual cell bursts, **(D)** the number of network bursts, and **(E)** the synchrony index. Data are expressed as the mean ± SEM (*n* = 3–6) and analyzed using the Kruskal–Wallis test; *p ≤ 0.05, **p ≤ 0.01, ***p ≤ 0.001. These experiments were conducted on cells between 16- and 20-weeks neural differentiation.

4-AP induces epileptiform-like activity both *in vivo* and *in vitro* (Peña and Tapia, 2000; Heuzeroth et al., 2019). In pilot experiments we established that 4-AP (10–100 μM) dose-dependently increased mean firing rate (Figure 6B). Moreover, 4-AP (100 μM) triggered single electrode bursts (*ictal* events, Figure 6C), network bursts (Figure 6D) and highly synchronized firing across our stem cell derived neuroglia cultures (Figure 6E). These events mirror epileptiform firing that were rare in unprovoked cultures at 16–20 weeks differentiation (the timing for this series of experiments). In subsequent experiments we therefore determined the impact of the five antiseizure agents against 4-AP (100 μM) induced changes in mean firing rate, single electrode bursts, network bursts and synchronized firing.

**Figure 7:**
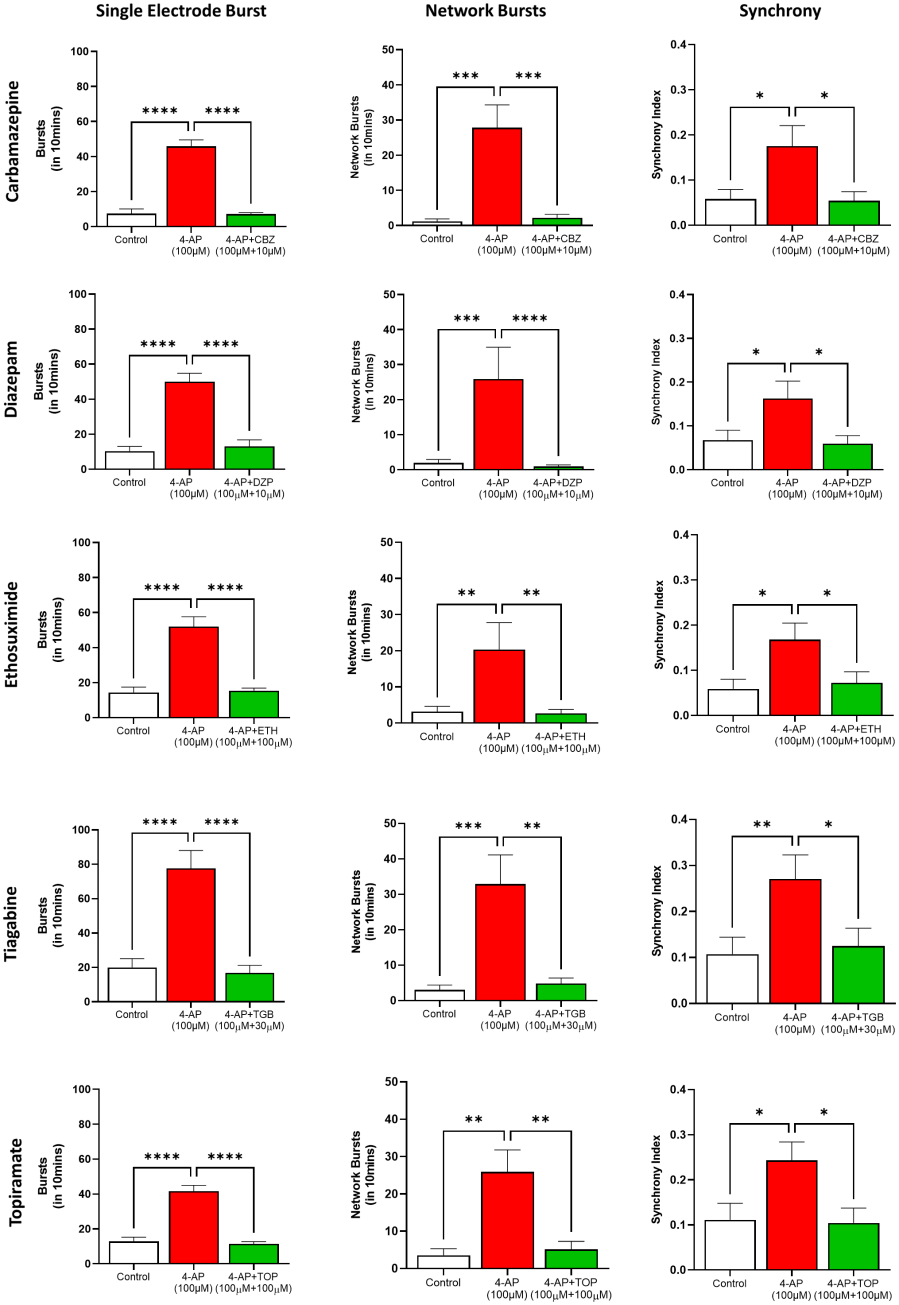
Inhibition of 4-AP evoked epileptiform neural activity in human TERA2.cl.SP12 stem cells derived neurons by antiseizure agents: the histograms show 4-Aminopyridine (4-AP, 100 μM) evoked increases in cell burst firing **(left column)**, network bursting **(central column)** and synchronized firing **(right column)** and, from top to bottom, the inhibitory effects of carbamazepine (10 μM), diazepam (10 μM), ethosuximide (100 μM), tiagabine (30 μM), and topiramate (100 μM). Data are expressed as the mean ± SEM (*n* = 6–9) and analyzed using the Kruskal–Wallis test; *p ≤ 0.05, **p ≤ 0.01, ***p ≤ 0.001, ****p ≤ 0.0001. Experiments were conducted on cells between 16- and 26-weeks neural differentiation.

Diazepam (10 μM) and carbamazepine (10 μM) each reduced the spike rate, single cell bursts and synchrony index back to control levels and completely abolished 4-AP (100 μM) induced network bursts. Similarly, topiramate (100 μM), ethosuximide (100 μM) and tiagabine (30 μM) each reversed the 4-AP-(100 μM) induced epileptiform spike, single cell bursts, network bursts and synchrony back to baseline levels (Figure 7). Together, these data show that our long-term human stem cell-derived neuroglia cultures and circuits express many of the molecular targets for a broad range of antiseizure agents.

## Discussion

Modeling the complex and prolonged development of the human nervous system *in vitro* is a profound challenge. Many experimental studies utilize human stem cell-derived neurons within days to weeks of neural differentiation that are unlikely to represent mature neural phenotypes (Halliwell, 2017), or contain glial cells, or to have formed synaptically connected neural circuits (Sharlow et al., 2020). To circumvent these significant limitations, some have utilized mixed populations of *induced* neurons and co-cultured them with human primary astrocytes to generate functional neuroglial cultures (e.g., Saavedra et al., 2021). In the current study we used TERA2.cl.SP12, a single human pluripotent stem cell line, with a simple and robust protocol, to differentiate both neurons and glial cells and characterized their development and functional maturation over 1 year in culture. From immunolabeling, we determined that neurons began to appear within 2 weeks of the start of neural differentiation and continued to expand for at least 3 months, as previously reported (e.g., Coyne et al., 2011; Cao et al., 2015). Our immunoblotting of the neural protein, β-III tubulin expression showed strong expression from 2 months through to 12 months in culture, indicating stable and prolonged viability of neurons. Glial cells appeared around 50 days from the start of differentiation, according to our immunolabeling, and from our GFAP protein expression data, greatly increased at 4 and 8 months in cell culture and showing that gliogenesis followed neurogenesis *in vitro*. These observations also show that neural differentiation of human EC stem cells, like human ES and iPS cells, follow an intrinsic developmental program and a timescale paralleling that seen *in vivo* (Itsykson et al., 2005; Johnson et al., 2007; Hu et al., 2010; Liu and Zhang, 2011; Nicholas et al., 2013; Paavilainen et al., 2018).

Our MEA recordings revealed the prolonged time course of 7–9 months for physiological maturation of human stem cell-derived neurons, and the establishment of inhibitory and excitatory synapses and neural circuit formation *in vitro*. Thus, neural spiking was absent for the first 2 weeks, but low frequency (≤0.5 Hz) firing developed over weeks 2–8. These observations are consistent with single cell recordings from these neurons showing that few are spontaneously active during the early phase of neural differentiation and only able to generate single, immature action potentials when stimulated with injection of depolarizing current (Coyne et al., 2011; Cao et al., 2015). However, the neural spike rate, the number of active cells and those able to generate bursts of action potentials slowly increased between weeks 12 and 28, closely following the first appearance and subsequent expansion of astrocytes from 8 to 32 weeks in the cultures. Thereafter, single cell bursts, synchronized network firing and oscillatory rhythms, greatly increased and plateaued around 46 weeks in culture and correlated well with the expansion of glial cells and the marked increase in synapse formation, as indicated by the expression of synaptophysin. Our data therefore support the observation that increased neurophysiological activity is associated with glial cell promotion of neuronal maturation, axonal elongation and synapse formation (Tang et al., 2013; Kayama et al., 2018; Hyvärinen et al., 2019; Hedegaard et al., 2020).

Human ES and iPS cell derived neurons *in vitro* show a similar developmental chronology but require co-culture with rodent or human astrocytes and show limited levels of bursting, low synchrony and electrical activity diminishes in longer term culture (Odawara et al., 2016; Kayama et al., 2018; Hedegaard et al., 2020; Kreir et al., 2022). Moreover, Jezierski et al. (2022) in *benchmarking* human amniotic fluid iPSC-derived *iNeurons* reported that they did not create a functional neural network or exhibit properties of fully mature neurons. In contrast, the TERA2.cl.SP12 stem cell derived neuroglia cultures described here recapitulated, over 12 months, the chronological sequence and physiological maturation of neural development observed *in vivo* (Khazipov and Luhmann, 2006; Kohler et al., 2011; Moore et al., 2011; Nicholas et al., 2013) and notably did not show diminished activity, even at 1 year *in vitro*.

Electrophysiological activity in our stem cell derived neuroglial cultures was modulated by a diverse array of voltage and ligand-gated ion channel agents. For example, the sodium channel inhibitor, TTX, and the potassium channel activator, retigabine, reduced spiking, consistent with voltage-activated action potentials. The effects of these control agents acting at sodium or potassium channels were consistent across the year in culture. The GABA_A_ antagonists, bicuculline and picrotoxin, increased spike rates, whereas the GABA_A_ allosteric potentiators, diazepam and phenobarbital, decreased firing, consistent with inhibitory synapses mediated by GABA_A_ receptors. Similarly, the broad-spectrum glutamate receptor antagonist, kynurenic acid, the selective AMPA receptor antagonist, DNQX, and the selective NMDA receptor blocker, D-AP5, reduced network activity in the cultures showing that excitatory neurotransmission is mediated by ionotropic glutamate receptors. The inhibitory actions of the GABA_A_ and the glutamate receptor antagonists increased as the cells matured *in vitro*, indicating an increased number of active inhibitory and excitatory synapses in our neuroglial cell cultures, and consistent with that observed in hiPSC-derived neurons in long-term culture (Odawara et al., 2016). Together, our MEA electrophysiology and neuropharmacology data, over ultra-long-term culture, show the development of a heterogeneous population of mature human neurons, supported by glia, that form inhibitory and excitatory synapses and mediate neurotransmission through adherent 2D circuits. Additional work, however, is required to further characterize the identity of the neuron subtypes in these functionally complex cell cultures.

Six widely used anticonvulsant agents, carbamazepine, diazepam, ethosuximide, tiagabine, topiramate and valproic acid, with diverse mechanisms of action (Sills and Rogawski, 2020; Hakami, 2021), reduced spontaneous spike activity in our neuroglia cultures, but it was not changed by the negative control drugs, caffeine, acetaminophen or indomethacin. These data show selective sensitivity to neurologically active medications and support the conclusion that we have functionally mature neurons and 2D neural circuits *in vitro*.

The proconvulsant, 4-aminopyridine (4-AP), robustly evoked increases in action potential frequency, single cell burst firing, and synchronized network bursting in our human EC stem cell derived neuroglial cultures, emulating epileptiform activity, and consistent with primary rodent brain slice and cultured rat cortical neurons data (e.g., Heuzeroth et al., 2019; Tukker and Westerink, 2021). In contrast, two recent studies reported that 4-AP did not induce epileptiform activity in several iPSC derived neuron lines, including *iNeurons*, *iCell Glutaneurons* or *CNS.4 U* neuron cultures (Tukker et al., 2020; Jezierski et al., 2022) suggesting they do not fully replicate the cellular properties of mature neurons or neural circuit complexity. Several reasons for their insensitivity to 4-AP include the *maturity* of these neurons, the cell composition (e.g., absence or presence of glia) of the cultures (e.g., Jezierski et al., 2022) but represent critical questions when validating a neuroglial network for *in vitro* disease modeling and/or drug discovery.

To our knowledge, the antiseizure drugs evaluated in this study represent the most diverse group of clinically important agents tested against mature human stem cell-derived neurons in ultra-long term cultures; they target an array of molecular sites in the central nervous system including voltage-gated sodium channels (carbamazepine), T-type calcium channels (ethosuximide), the GABA transporter 1 (tiagabine), the benzodiazepine allosteric site on GABA_A_ receptors and pleiotropic actions on ionotropic glutamate receptors, GABA transmission, sodium channels and T-type calcium channels (topiramate) (reviewed by Sills and Rogawski, 2020). These anticonvulsants reduced, or completely blocked 4-AP evoked epileptiform-like activity in our human neuroglial cell cultures at concentrations close to, or within those measured in human serum following therapeutic dosing for seizure control (Hakami, 2021). Our findings therefore indicate these molecular targets are well expressed in our stem cell-derived neurons.

Kreir et al. (2022) reported that the first generation antiseizure agent, phenytoin reduced network burst duration recorded from hiPSC (CNS.4 U) derived neuron cultures (Kreir et al., 2022); similarly, Autar et al. (2022) demonstrated that valproic acid reduced bicuculline evoked increased firing recorded from cultures of hiPSC-derived cortical neurons (devoid of astrocytes) (Autar et al., 2022). These data are consistent with an earlier study of rat hippocampal neurons cultured on MEA plates showing that spontaneous and bicuculline-evoked firing could be modulated by high concentrations of carbamazepine and to some extent valproic acid (Colombi et al., 2013). A study utilizing human iPSC-derived neurons co-cultured with iPSC-derived astrocytes reported that epileptiform activity induced by the convulsant, pentylenetetrazol (a GABA_A_ antagonist) could be suppressed by phenytoin and valproic acid (Odawara et al., 2016). In a subsequent study however, phenytoin paradoxically increased single cell burst firing induced by 4-AP in these cells and the reason remains unclear (Odawara et al., 2018).

Notwithstanding, human stem cells, because of their unique abilities to expand *ad infinitum* and differentiate into other cell types, provide a powerful option for disease modeling and drug investigations *in vitro* (Westerink, 2013; Tukker and Westerink, 2021). Current protocols use a variety of human stem cells, including neural stem cells that are not easily accessible, embryonic stem cells that are unacceptable in some communities, and induced pluripotent stem cells that require extensive de-differentiation and reprogramming protocols. In contrast, several EC stem cell lines are pluripotent with the capacity to differentiate into all three germ layers, are easy to grow in culture and do not require feeder cells. In this study, we have shown that using a very simple differentiation protocol, the human pluripotent stem cell line TERA2.cl.SP12 (derived from a single clone; Przyborski, 2001) differentiate *in vitro* into mature neurons and glia cells over 6–8 months, paralleling human neurogenesis *in vivo*; these neuroglia cultures display complex electrical activity including single cell and network burst firing, synchronized and rhythmical firing patterns across integrated neural circuits that are comprised of both inhibitory and excitatory synapses. Neural activity in our 2D neuron–glia circuits is modulated by a variety of voltage-gated ion channel agents and ligand-gated receptor drugs, as well as a diverse array of antiseizure agents, consistent with animal and human studies. Together, such observations support their use in disease modeling and neuropsychiatric drug discovery. The prolonged culture time to maturity can make experiments more challenging but it also provides opportunities to investigate the longer-term impact of gene mutations and drug exposures on early neural development. In addition, neurons are functional for over 1 year, providing extensive time for diverse neuropharmacology testing. Finally, utilization of differentiated neurons and glia over ultra-long-term culture may be a valuable *in vitro* representation of human neurons that naturally develop over 9 months *in utero* and continue to elaborate and function over an average of eight decades of human life.

## Data availability statement

The raw data supporting the conclusions of this article will be made available by the authors, without undue reservation.

## Author contributions

HS conducted the stem cell culture, microscopy, immunohistochemistry (IHC), immunoblotting, MEA recordings and most of the neuropharmacology experiments, and contributed to the analysis of all the data presented in the manuscript. AP helped to conduct the protein expression experiments and the data analysis. AR organized and conducted some of the immunoblotting experiments and the data analysis. BZ conducted some of the microscopy and IHC experiments and edited the manuscript. RH designed the study, conducted some of the cell culture and MEA recordings, analyzed all data, and wrote the first draft of the manuscript. All authors contributed to the article and approved the submitted version.

## Conflict of interest

The authors declare that the research was conducted in the absence of any commercial or financial relationships that could be construed as a potential conflict of interest.

## Publisher’s note

All claims expressed in this article are solely those of the authors and do not necessarily represent those of their affiliated organizations, or those of the publisher, the editors and the reviewers. Any product that may be evaluated in this article, or claim that may be made by its manufacturer, is not guaranteed or endorsed by the publisher.
